# Review of the grassland leafhopper genus *Exitianus* Ball (Hemiptera, Cicadellidae, Deltocephalinae, Chiasmini) from China

**DOI:** 10.3897/zookeys.333.5324

**Published:** 2013-09-20

**Authors:** Yani Duan, Yalin Zhang

**Affiliations:** 1 School of Plant Protection, Anhui Agricultural University, Hefei, Anhui Province 230036, China; 2 Key Laboratory of Plant Protection Resources and Pest Management of Ministry of Education, Entomological Museum, Northwest A & F University, Yangling, Shaanxi Province 712100, China

**Keywords:** Hemiptera, Auchenorrhyncha, morphology, taxonomy

## Abstract

The two Chinese species of the leafhopper genus *Exitianus* Ball (Hemiptera: Cicadellidae: Deltocephalinae: Chiasmini) (*Exitianus indicus* (Distant) and *Exitianus nanus* (Distant)) are reviewed. Descriptions of the species and a key for their separation are provided. *Exitianus fulvinervis* Li & He is considered a junior synonym of *Exitianus nanus* **syn. n.**

## Introduction

**Figure 1.**
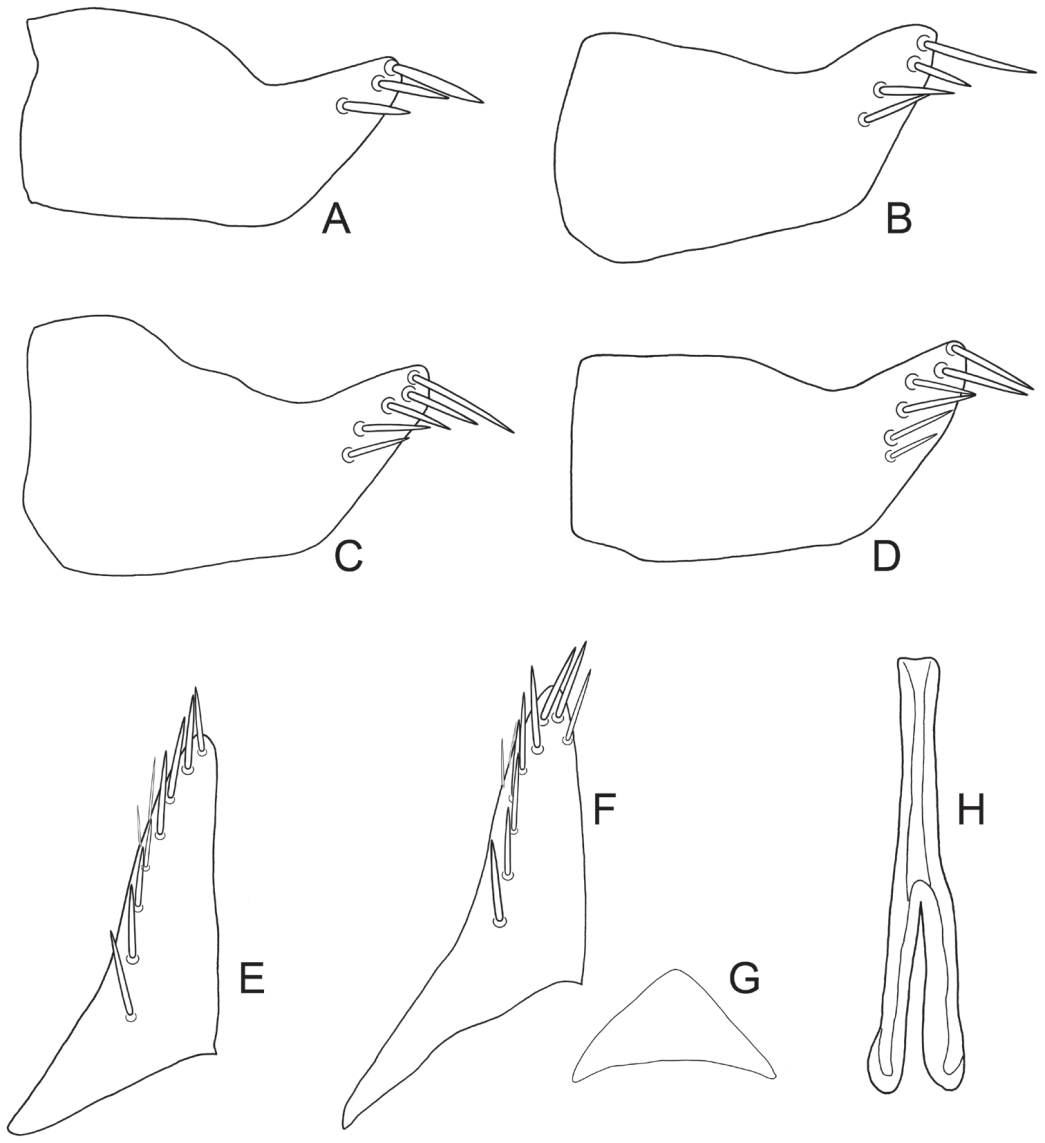
*Exitianus nanus*.**A–D** male pygofer side, lateral view **E–F** subgenital plate, ventral view **G** valve, ventral view **H** connective, dorsal view.

Among the most widespread and often abundant tropical and temperate species of grassland leafhoppers are the moderately large tawny forms comprising the genus *Exitianus* Ball. It contains 43 species of which 6 species occur in Asia. Members of the genus are most readily distinguished by usually having a transverse dark band on the vertex (Plate II: A–E), males with a small number of apical stout setae on the pygofer (Figs 1A–D) and the female with a relatively long ovipositor extending conspicuously beyond the last dorsal segment (Plate I: D). These characters are shared only by the presumably sister genus *Nephotettix* Matsumura, but which differs in having the crown sharply ridged where it meets the face, and being opaque green with various black markings.

This study reviews for the first time the species of *Exitianus* from China. From using the revision of Ross (1968) we identify two species (*Exitianus indicus* (Distant) and *Exitianus nanus* (Distant)) but have found and figured further variation in the male genitalia to that given by Ross for the two species. With respect to *Exitianus nanus* these findings allow us to place *Exitianus fulvinervis* Li & He, from China, as a junior synonym. As our work includes the study of numerous specimens we conclude that the remaining three species recorded from China, i.e., *Exitianus capicola* (Stål) by Matsumura (1914: 186), *Exitianus coronatus* (Distant) by Li et al. (2011: 69, fig. 57) and *Exitianus fusconervosus* (de Motschulsky) by Kuoh (1966: 143, fig. 133) are probably misidentifications.

## Material examined

Material examined is deposited in the Entomological Museum of Northwest A & F University (NWAFU) and the Institute of Zoology, the Chinese Academy of Sciences (IZCAS). Morphological terminology follows Oman (1949) and Zhang (1990).

## Taxonomy

### Exitianus

Ball

http://species-id.net/wiki/Exitianus

Exitianus Ball, 1929: 5. Type species: *Cicadula obscurinervis* Stål.
Mimodrylix Zachvatkin, 1935: 108. Type species: *Athysanus capicola* Stål. Synonymized by Evans 1947: 235.
Exitianus ; Ross 1968: 1–30 [Review].
Exitianus ; Oman et al. 1990: 213 [Listed; Athysanini].
Exitianus ; Fang et al. 1993 [Phylogeny, mitochondrial sequences].
Exitianus ; Emeljanov 1999: 547 [To Doraturini].
Exitianus ; Dmitriev 2003: 677 [Immatures].
Exitianus ; Zahniser and Dietrich 2013: 56 [To Chiasmini].

#### Remarks.

An adequate description of this genus is given by Ross (1968). See introduction for the main distinguishing features. The two species from China can be separated by the following key.

#### Key to species of *Exitianus* Ball from China

**Table:** 

1	Vertex usually with transverse arcuate brown band interrupted medially (Plate I: A–B). Scutellum with dark brown basal triangles (Plate I: A–B). Male pygofer side with 2–6 apical brown or black macrosetae (Figs 1A–D). Aedeagal shaft slightly laterally compressed with small gonoduct (Fig. 2C); without processes (Figs 2B–C). Female VIIth sternite with posterior margin tri-lobed (Plate I: D)	*Exitianus nanus*
–	Vertex usually with transverse arcuate brown band complete (Plate II: A–E). Scutellum with faint brown basal triangles (Plate II: A–E). Male pygofer side with 2–3 apical brown or black macrosetae (Figs 3–4). Aedeagal shaft strongly laterally compressed, gonopore large with rim forming concave margin in lateral view; with pair of small dorsobasal processes (Figs 5G, 6). Female VIIth sternite with a shallow notch in mid-line (Plate II: G).	*Exitianus indicus*

**Plate I.**
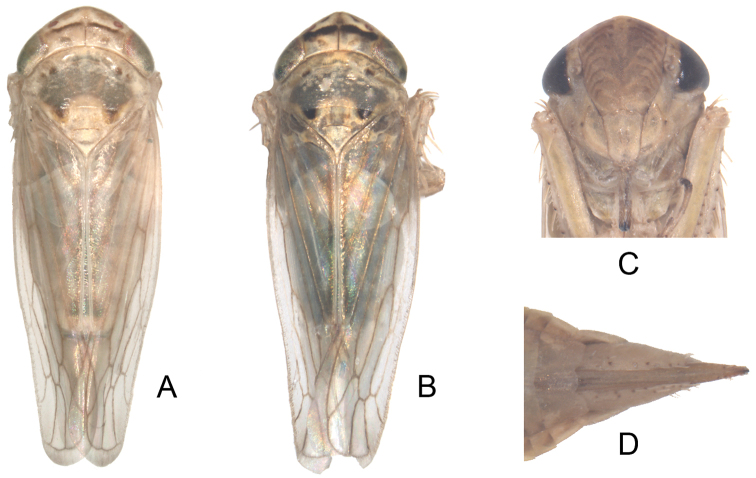
*Exitianus nanus*.**A–B** habitus, dorsal view **C** face **D** the end of female abdomen, ventral view.

### Exitianus nanus

(Distant)

http://species-id.net/wiki/Exitianus_nanus

**Figure 2.**
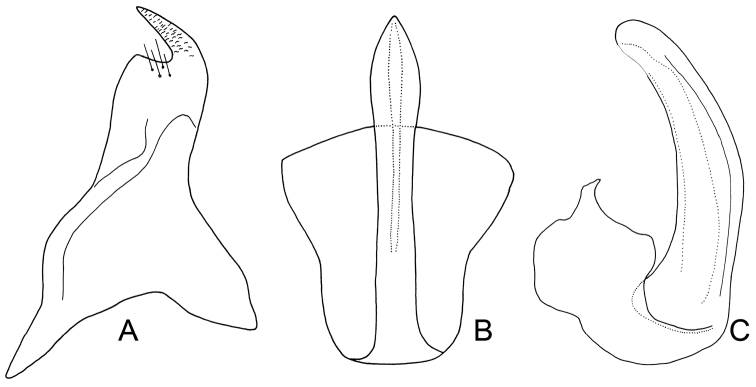
*Exitianus nanus*.**A** style, dorsal view **B, C** aedeagus, ventral and lateral view, respectively.

Plate I , Figures 1 –2

Athysanus nanus Distant, 1908: 345.
Athysanus insularis ; Distant 1909: 47, pl. 4, figs 10, 10a. Synonymized by Ross 1968: 7.
Athysanus fasciolatus ; Melichar 1911: 107. Synonymized by Linnavuori 1975: 626.
Athysanus simillimus ; Matsumura 1914: 185. Synonymized by Ross 1968: 7.
Athysanus vulnerans ; Bergevin 1925: 42, figs 5–9. Synonymized by Ross 1968: 7.
Limotettix albipennis ; Haupt 1927: 25, pl. II, figs 20a–c.Synonymized byDlabola 1963: 325.
Limotettix unifasciata ; Haupt 1930: 159, fig. 9. Synonymized byDlabola 1963: 325.
Athysanus digressus ; Van Duzee 1933: 32. Synonymized by Linnavuori and DeLong 1978: 237.
Exitianus nanus ; Ross 1968: 7, figs 1–3,15–18, 76.
Exitianus karachiensis ; Ahmed 1986: 59, fig. 5. Synonymized by Khatri and Webb 2010: 10.
Exitianus peshawarensis ; Ahmed and Rao 1986: 76–77, fig. 1. Synonymized by Khatri and Webb 2010: 10.
Exitianus minor ; Ahmed et al. 1988: 12, fig. 2. Synonymized by Khatri and Webb 2010: 10.
Exitianus fulvinervis ; Li and He 1993: 27; Li et al. 2011: 68, fig. 55. **syn. n.**

#### Description.

Length. Male: 3.0–4.1mm; female: 3.9–5.2mm.

Yellow-brown with variable brown markings on vertex comprising spots on anterior margin and a more posterior arcuate band interrupted medially (Plate I: A–B); frontoclypeus with faint brown lateral arcs (Plate I: C). Pronotum usually with some dark infuscation (Plate I: A–B). Scutellum with dark brown basal triangles (Plate I: A–B).

Crown width about 3× length (Plate I: A–B).

**Male genitalia.** Pygofer side usually with 2–6 apical brown or black macrosetae (Figs 1A–D). Style preapical lobe broadly triangular, apophysis evenly tapered to apex (Fig. 2A). Aedeagal shaft slightly laterally compressed with small subapical dorsal gonoduct (Fig. 2C); without processes (Figs 2B–C).

**Female.** Sternite VII with posterior margin tri-lobed (Plate I: D).

#### Material examined.

**China: Zhejiang Prov.:** 1 female, Wuyanling, 2.VIII.2005, coll. DY; **Fujian Prov.:** 8 males, 15 females, Wuyishan, Nantan, 12.VIII.2002, coll. SQ; 41 males, 48 females, Guangze County, 23.VIII.2002, coll. SQ; **Jiangxi Prov.:** 16 males, 5 females, Anfu County, 10.VIII.2002, coll. SQ; **Henan Prov.:** 1 male, Baiyunshan, 17.VIII.2008, coll. LL; **Hunan Prov.:** 1 female, Hengshan, 30.VIII.1980, coll. TX; **Guangdong Prov.:** 1 male, 1 female, Shenzhen City, VIII.1986, coll. ZY; **Guangxi Prov.:** 1 female, Fangcheng City, 3.VI.2000, coll. Li Wenzhu (IZCAS); **Hainan Prov.:** 10 males, 2 females, Liangyuan, 10/29.V.1983, coll. ZY; 1 male, Wuzhishan, 640m, 16.V.2007, coll. DY; 2 males, 5 females, Jianfengling, 22.VII.2010, coll. WY; 4 males, Wuzhishan City, Maoyang Town, 3.VIII.2010, coll. Sun Jing; 3 females, Yinggeling, 4.VIII.2010, coll. WY; 30 males, 40 females, Bawangling, 9.VIII.2010, coll. WY; 7 males, 2 females, Limu, 933m, 22.VIII.2010, coll. WY; 61 males, 30 females, Tongguling, 26.VIII.2010, coll. WY; **Yunnan Prov.:** 1 male, Mengla County, Yaoqu Town, 660m, 3.V.1991, coll. Liu Guanchun & Cai Wanzhi; 1 male, Mengla County, Yaoqu Town, 18.VI.1991, coll. WT; 2 females, Mengla County, Menglun, 19.V.1991, coll. WT; 2 males, Jinghong City, Jinghong County, 30.VIII.2010, coll. Han Juan; 1 male, Jinghong City, Jinghong County, 23.X.2010, coll. Chai Yonghui & Feng Jinian; 1 male, Menglian County, 971m, 24.V.2011, coll. LL; 1 male, Lancang County, 25.V.2011, coll. LL; 2 males, Yexianggu, 1226m, 9.VI.2011, coll. LL; 1 male, Zhenyuan County, 13.VI.2011, coll. LL. All deposited in NWAFU, except where indicated and mainly collected at light. Abbreviations for collectors: DY: Duan Yani; SQ: Sun Qinxia; LL: Lu Lin; TX: Tong Xinwang; ZY: Zhang Yalin; WY: Wang Yang; WT: Wang Yinglun & Tian Rungang.

#### Distribution.

Eastern Hemisphere.

#### Remarks.

*Exitianus fulvinervis* was described by Li & He (1993) based on specimens collected from Tibet. As these fall within the variation found in *Exitianus nanus*, the two species are here synonymised.

### Exitianus indicus

(Distant)

http://species-id.net/wiki/Exitianus_indicus

**Figure 3.**
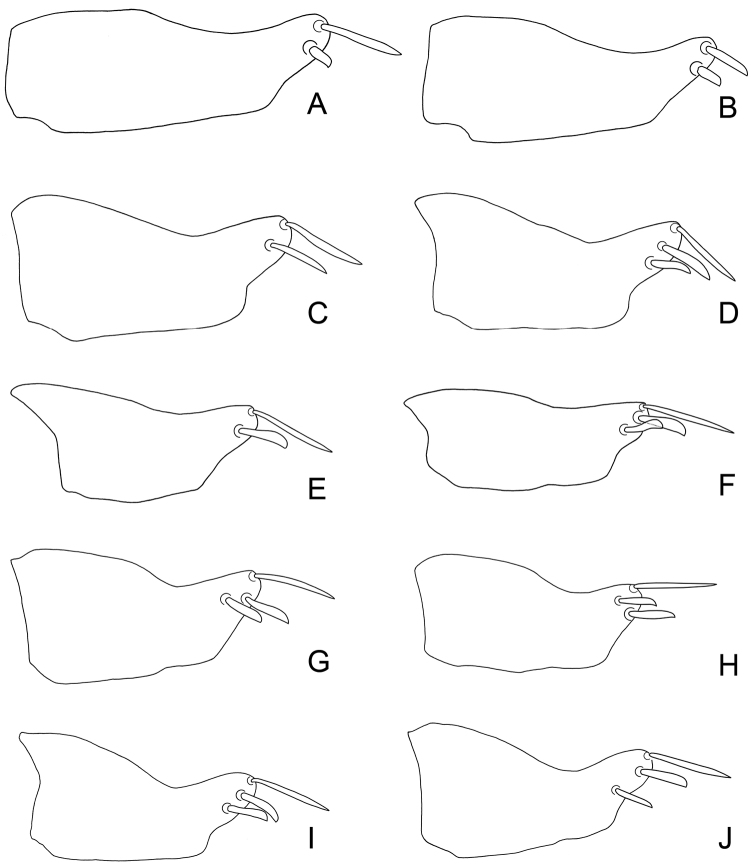
*Exitianus indicus*. **A–J** male pygofer side, lateral view from the same specimen, respectively.

Plate II , Figures 3 [Fig F6] [Fig F7] –6

Athysanus indicus Distant, 1908: 344.
Athysanus atkinsoni ; Distant 1908: 345. Synonymized by Ross 1968: 12.
Exitianus indicus ; Ross 1968: 12, figs 9–10,26–30, 69.
Exitianus major ; Ahmed et al. 1988: 10, fig. 1. Synonymized by Khatri and Webb 2010: 10.

#### Description.

Length. Male: 4.0–4.5mm; female: 4.0–5.2mm.

Yellow-brown with variable transverse arcuate brown band on vertex (Plate II: A–E); frontoclypeus with faint brown lateral arcs (Plate II: F). Scutellum with faint brown basal triangles (Plate II: A–E).

**Figure 4. F6:**
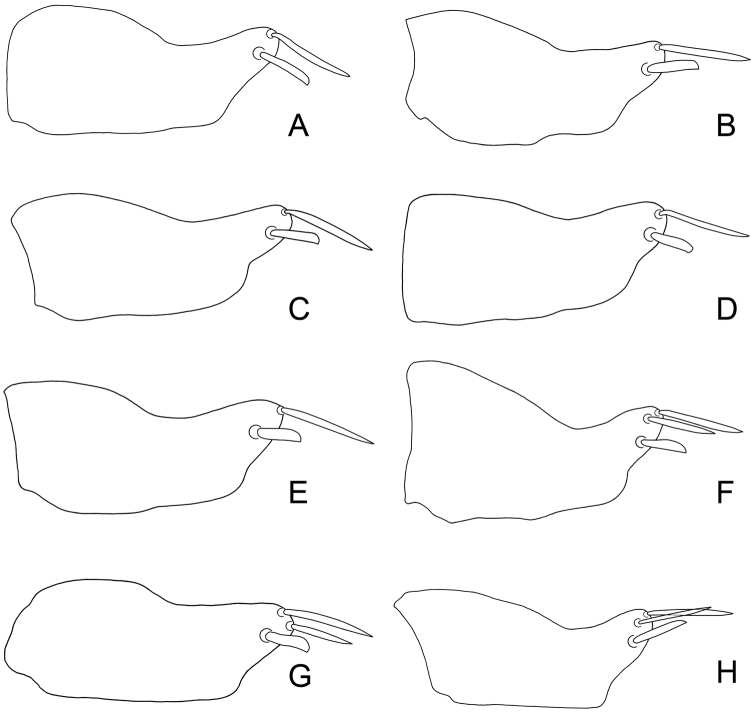
*Exitianus indicus*. **A–H** male pygofer side, lateral view.

**Figure 5. F7:**
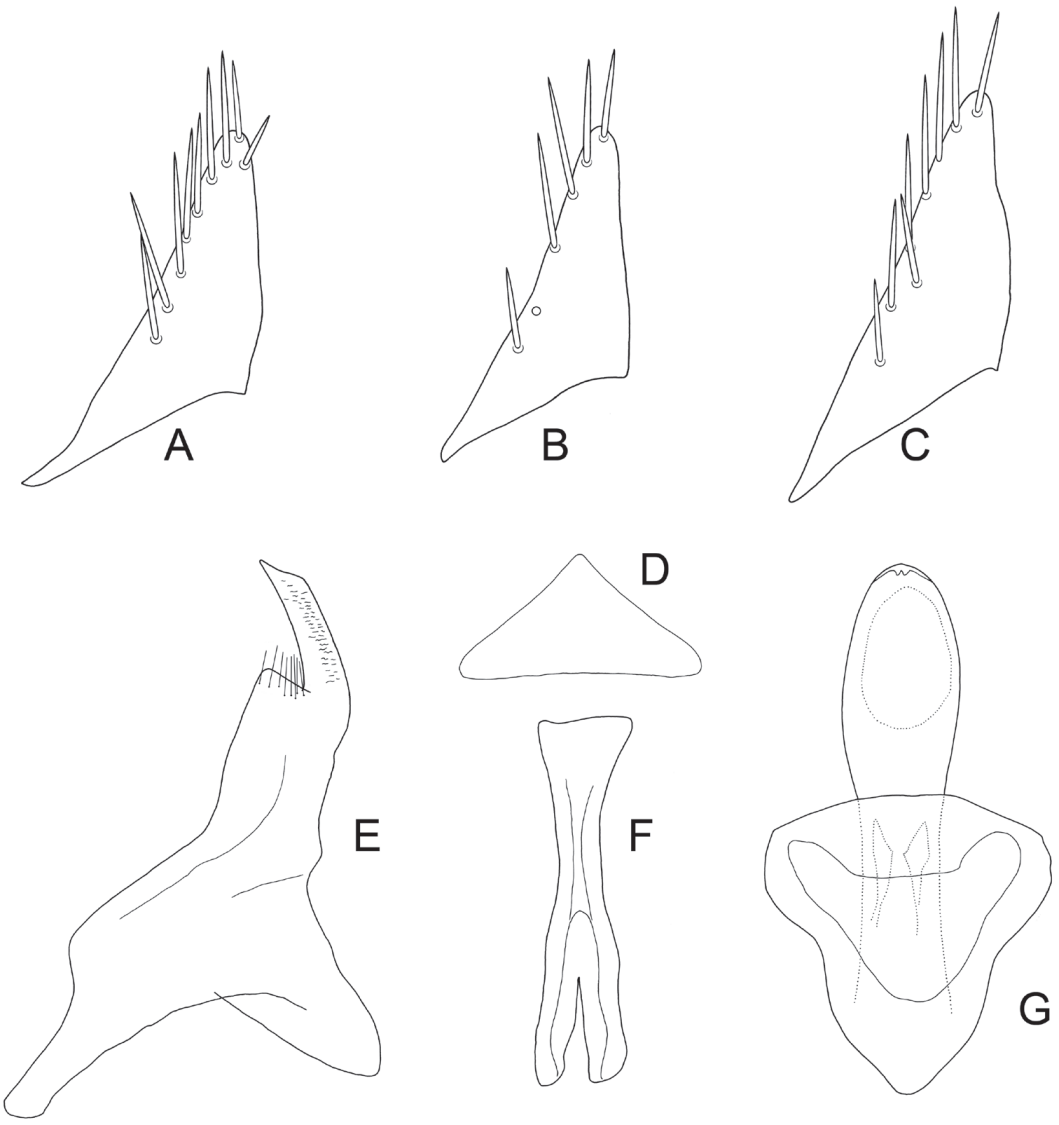
*Exitianus indicus*. **A–C** subgenital plate, ventral view **D** valve, ventral view **E** style, dorsal view **F** connective, dorsal view **G** aedeagus, dorsal view.

**Figure 6.**
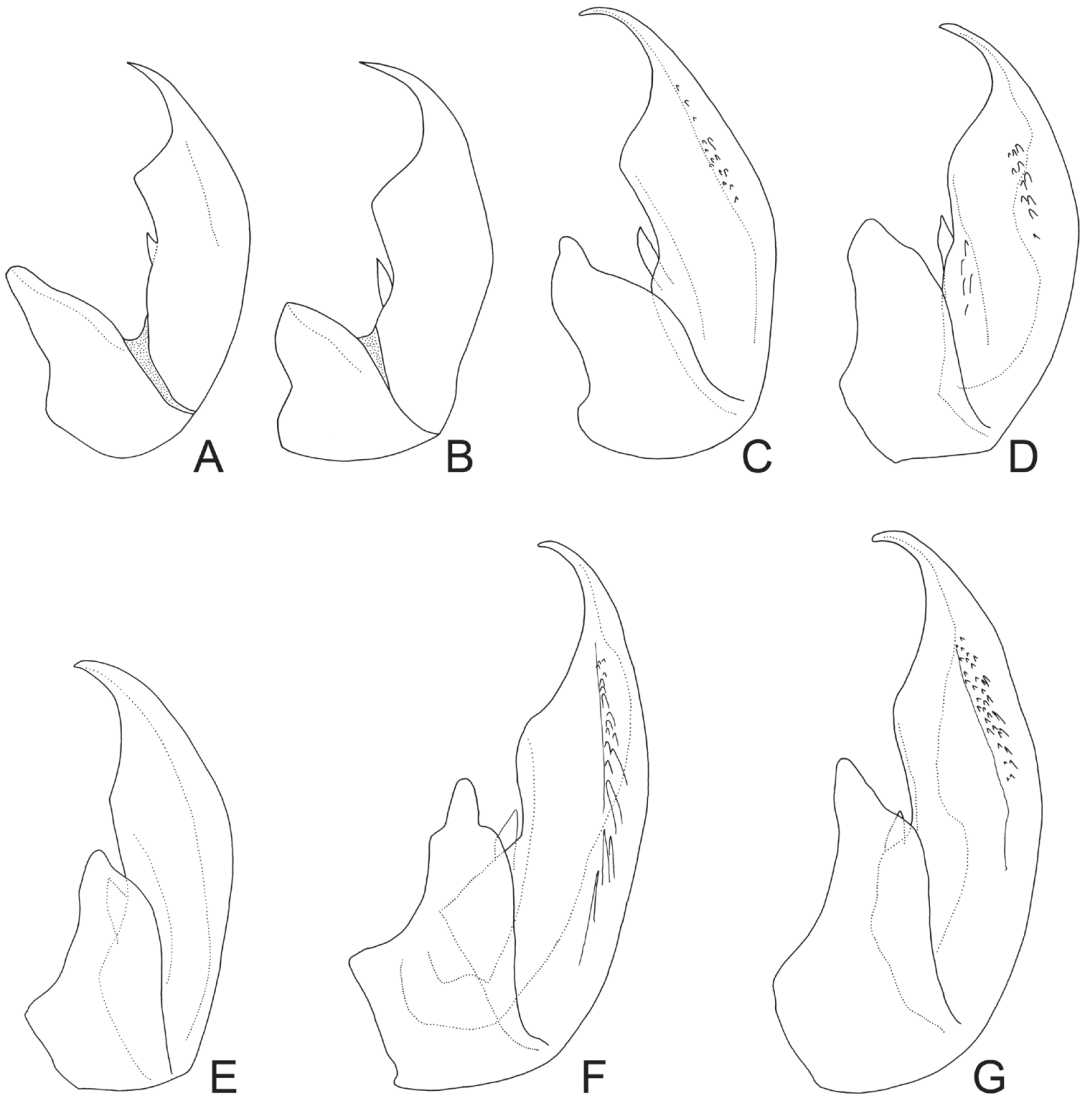
*Exitianus indicus*. **A–G** aedeagus, lateral view **A, B** (after Ross, 1968)

**Male** **genitalia.** Pygofer side usually with 2–3 apical brown or black macrosetae (Figs 3–4). Style preapical lobe narrowly triangular, apophysis abruptly tapered at apex (Fig. 5E). Aedeagal shaft strongly laterally compressed, gonopore large with rim forming concave margin in lateral view; with pair of small dorsobasal processes (Figs 5G, 6).

**Female.** Sternite VII with posterior margin with a shallow notch in mid-line (Plate II: G).

**Plate II.**
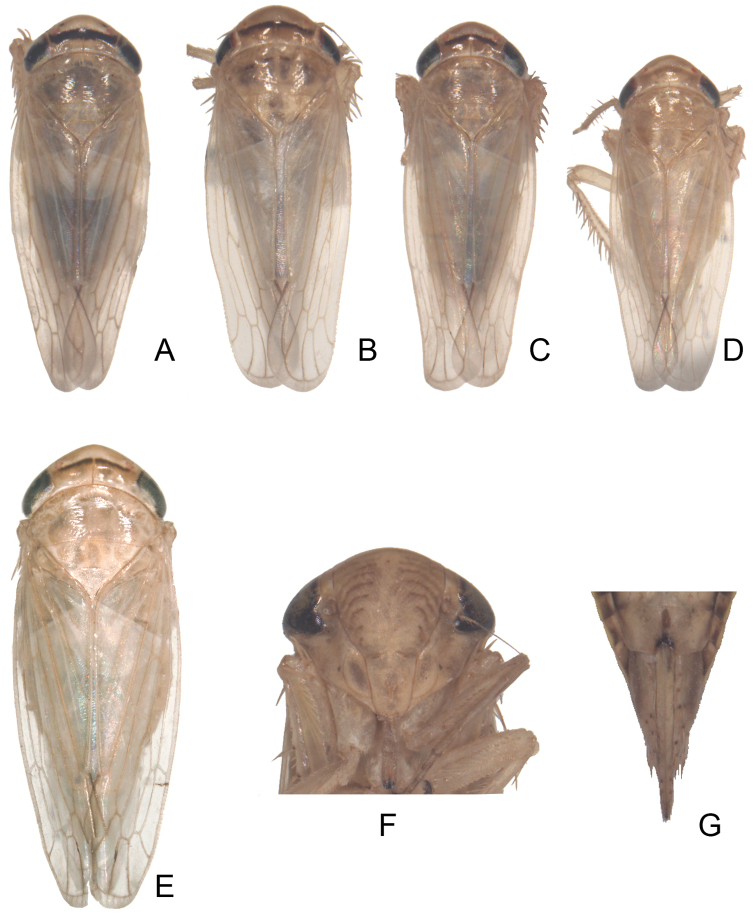
*Exitianus indicus*.**A–E** habitus, dorsal view **F** face **G** the end of female abdomen, ventral view.

#### Material examined.

**China: Jilin Prov.:** 1 male, Lingjiang City, 30.VII.1983, coll. Wu Zhengliang & Hua Baozhen; **Zhejiang Prov.:** 21 males, 10 females, Wuyanling, 400m, 1.VIII.2005, coll. DY; **Fujian Prov.:** 1 male, Wuyishan, Nantan, 18.VIII.2002, coll. SQ; 2 males, Guangze County, 24.VIII.2002, coll. SQ; 1 male, Wuyishan Sanggang, 19.VII.2006, coll. YM; 41 males, 10 females, Shanghang County, Buyun Town, 21.VII.2009, coll. CY; 3 males, 1 female, Wuyishan, Tongmucun, 7.VIII.2009, coll. CY; **Jiangxi Prov.:** 34 males, 10 females, Anfu County, 10.VIII.2002; 4 males, 4 females, Ruijin City, Baying Town, 280m, 15.VIII.2004, coll. WC & YM; 7 males, 6 females, Pingxiang City, Lianhua County, 4.VIII.2002, coll. SQ; **Henan Prov.:** 1 male, Jigongshan, 11.VII.1997, coll. SQ; 1 male, Ruijin City, Baying Town, 280m, 15.VIII.2004, coll. WC & YM; 1 male, Baiyunshan, 17.VIII.2008, coll. LL; **Hubei Prov.:** 1 male, Wudangshan, 22.VII.2001, coll. Huang Min & Zhang Guiling; **Hunan Prov.:** 1 male, Chenzhou City, 28.VIII.1985, coll. ZY & Chai Yonghui; 9 males, 3 females, Changde City, Huanan Factory, 22.VII.2002, coll. SQ; 6 males, Zhangjiajie, 25.VII.2002; **Guangdong Prov.:** 1 male, Dianbai County, 13.IV.1983, coll. ZY; 1 male, Shenzhen, 18.IV.1983, coll. ZY; 1 male, Dinghushan, 7.VII.1985, coll. ZY; **Guangxi Prov.:** 10 males, 4 females, Huaping, 9.VIII.2000, coll. LZ; 1 male, Huaping, 26.VIII.2000, coll. LZ; 5 males, Fangchenggang City, Naqin Town, 1–3.VIII.2001, coll. He Zhiqiang; 13 males, 6 females, Xinzhai, 18.VIII.2005, coll. YM & KJ;1 male, Yuanbaoshan, 12.VIII.2006, coll. YM & KJ; **Hainan Prov.:** 10 males, 5 females, Yacheng, 6.V.1983, coll. ZY; 1 male, Wuzhishan, 720m, 31.VII.2009, coll. Gaoxia; 1 male, Bawangling, 9.VIII.2010, coll. WY; 1 male, Tongguling, 26.VIII.2010, coll. WY; **Sichuan Prov.: **2 males, 9 females, Kangding, 2500m, 8.XI.1999, coll. Qin Daozheng; 2 males, 8 females, Zhubalong, 2450m, 11.VII.2011, coll. Sun Qiang; **Guizhou Prov.:** 5 males, Guiyang City, Huaxi Park, 1100m, 25.VII.2001; **Yunnan Prov.:** 1 male, Daluo Town, 26.X.1987, coll. Feng Jinian & Lili; 10 males, 8 females, Mengla County, Yaoqu Town, V.1991; 1 male, Tengchong County, 22.XI.1999; 2 males, Xishuangbanna, 11.VII.2003; 1 male, Mengla City, 21.VII.2005, coll. LL; 1 male, Simao City, 30.VII.2005, coll. LL; 31 males, 20 females, Tengchong County, Huoshan Park, 1930m, 14.VIII.2005, coll. YM & KJ; 1 male, Dali, 29.VIII.2010, coll. ZM;1 male, Diqing, 14.VIII.2010, coll. ZM; 1 male, Jinghong City, 1.IX.2010, coll. ZM; 1 male, Daluo Town, 679m, 23.V.2011, coll. LL; 1 male, Lancang County, 25.V.2011, coll. LL; 1 male, Nansan Town, 29.V.2011, coll. LL; 1 male, Yingjiang County, Zhanxi Town, 1009m, 2.VI.2011, coll. LL; 1 male, Tengchong County, 1632m, 5.VI.2011, coll. LL; 4 males, Lushui County, Chenggan Town, 1013m, 6.VI.2011, coll. LL; 1 male, Zhenyuan County, 13.VI.2011, coll. LL; **Shaanxi Prov.:** 1 male, Liuba County, 20.VII.1995, coll. ZR; 3 males, 3 females, Liuba County, 20.VIII.1995, coll. ZR; 1 male, Zhouzhi County, 24.IX.2008, coll. LL; 1 male, Foping County, 1060m, 1.X.2008, coll. LL; **Gansu Prov.:** 1 male, Cheng County, 25.VII.2002, coll. WC & Shang Suqin; 1 male, Ruijin City, Baying Town, 280m, 15.VIII.2004, coll. WC & YM. All deposited in NWAFU and mainly collected at light. Abbreviations for collectors: DY: Duan Yani; SQ: Sun Qinxia; LL: Lu Lin; YM: Yang Meixia; CY: Cao Yanghui; ZY: Zhang Yalin; ZM: Zhang Meng; ZR: Zhang Wenzhu & Ren Liyun; WC: Wei Cong; LZ: Liu Zhenjiang; KJ: Kang Juxia; WY: Wang Yang.

#### Distribution.

Eastern Hemisphere.

#### Remarks.

Considerablevariation was found in the apical black macrosetae of the male pygofer and shape of the aedeagus in this species (see Figs 3, 4 & 6).

## Supplementary Material

XML Treatment for Exitianus

XML Treatment for Exitianus nanus

XML Treatment for Exitianus indicus
